# Poisons Formed from Food

**Published:** 1886-07

**Authors:** 


### POISONS FORMED FROM FOOD.

The subject of "Poisons formed from Food, and their Relation to Biliousness and Diarrhœa," has been considered by Dr. T. Lauder Brunton in articles in The Practitioner. There are persons, he says, or even, perhaps, "classes of people," to whom even articles of food, usually salutary, are poisonous. Many articles of food, also, have a property of splitting themselves up so as to yield poisons. The melon and cucumber tribe of vegetables exhibit a tendency to the formation of purgative substances. In animal foods poisonous properties are apt to appear either from particular modes of cooking, or from beginning decomposition. The decomposition may be effected by microbic organisms, or by the digestive ferments of the healthy body; and they are various according to the peculiar organism or ferment that sets them up, and according to the temperature at which they occur, and the length of time that they continue. Some of the products of decomposition are poisonous in various degrees of activity, while others are innocuous. When kept separate, the poisonous products remain unchanged for a long time, but when mixed together they are apt to undergo further decomposition and become active. Besides temperature, the degree of moisture in the subject of decomposition or in the atmosphere, and electrical conditions—as when milk is "soured by thunder"—exercise modifying influences, which have not yet been definitely ascertained. The difference between the products of decomposition in hot and cold weather is illustrated by the alkaloids obtained by decomposing maize in summer and winter. The winter alkaloid has a narcotic and paralyzing action; but in summer another alkaloid is also yielded, which has a tetanizing action something like strychnine. On account of the greater rapidity of the putrefactive process, albuminous substances become poisonous much sooner in summer than in winter, and again lose their poisonous properties more quickly by further decomposition. As putrefaction may go on to a certain extent after the introduction of food into the intestinal canal, poisons may be formed from the part eaten, and produce dangerous symptoms, while no poison can be found in the remaining parts of the same food.

